# The school-led *Preventure* study: Protocol of a cluster-randomised controlled trial of effectiveness to prevent adolescent alcohol misuse, internalising problems, and externalising problems through a personality-targeted intervention delivered by school staff

**DOI:** 10.1016/j.pmedr.2020.101286

**Published:** 2020-12-19

**Authors:** Erin Veronica Kelly, Lucinda Rachel Grummitt, Louise Birrell, Lexine Stapinski, Emma Louise Barrett, Julia Boyle, Maree Teesson, Nicola Clare Newton

**Affiliations:** The Matilda Centre for Research in Mental Health and Substance Use, Sydney Medical School, The University of Sydney, Australia

**Keywords:** Prevention, Alcohol use, Mental health, Personality, School, Randomised controlled trial, Research translation

## Abstract

Mental disorders and problematic alcohol use are common, co-occurring and cause significant harm to individuals and society. It is critical to intervene early to prevent chronic and debilitating trajectories. Existing prevention programs among adolescents are limited in effectiveness and implementation. This Australian-first study will examine the effectiveness and feasibility of a personality-targeted program called Preventure, in preventing the onset or escalation of alcohol use, internalising problems and externalising problems among young Australians, when delivered by school staff.

A cluster randomised controlled trial (RCT) of effectiveness will be conducted from 2020 to 2022 with 12 schools in Sydney, Australia, with students aged 13 years at baseline. Schools will be randomly allocated to the *Preventure* intervention or a control condition who will receive their usual Health Education curriculum. Schools allocated to the intervention will deliver *Preventure* to students scoring one standard deviation above the population mean on one of four personality traits. *Preventure* consists of two 90-minute group sessions that incorporate cognitive-behavioural therapy and motivational interviewing to promote coping skills. Students will be invited to complete surveys at baseline, 6- and 12-months following the intervention. Primary outcomes include student alcohol use, internalising problems, and externalising problems. Implementation fidelity, feasibility and acceptability will also be examined through surveys with school staff and students. Ethical approval has been obtained from the University of Sydney Human Research Ethics Committee, and the State Education Research Applications Process for research in public schools in NSW. This trial is registered with the Australian New Zealand Clinical Trials Registry (ACTRN12620000790943).

## Highlights

• Australian-first school-led trial targets personality risk factors for psychopathology.
• Examines effectiveness for substance use, internalising, externalising outcomes.
• Seeks evidence for feasible and scalable delivery of *Preventure* for schools.
• Adds to limited research on effectiveness of prevention programs rather than efficacy.

## 1 Introduction

Mental disorders are the leading cause of disability for young people worldwide, accounting for 25% of all years lived with disability and over 1.3 million years of life lost (Vigo et al., 2016, Erskine et al., 2015). Mental disorders have significant social and economic impacts, largely attributable to their high prevalence, early age of onset, and chronic, disabling course. The most common mental disorders among adolescents in Australia are attention deficit hyperactivity disorder, anxiety disorders, depressive disorders, and conduct disorder (Lawrence et al., 2015). To make a significant impact on these disorders at a population level, it is imperative that public health shifts focus to prevention (Pine and Fox, 2015), rather than focusing on treating individuals with entrenched disorders.

Problematic alcohol use is also an issue of vital importance in Australia. While trends over the last 20 years show that Australian adolescents are delaying their onset of alcohol use, and are less likely to smoke tobacco and use illicit drugs, drinking at risky levels remains high (Australian Institute of Health and Welfare, 2019). Harmful alcohol use (exceeding the National Health and Medical Research Council guidelines (National Health and Medical Research Council, 2009) is a major health issue, associated with increased risk of chronic disease, injury and premature death (Australian Institute of Health and Welfare, 2019). Alcohol use is especially risky for adolescents given the impact on the developing brain and increased risk of being a victim of alcohol-related incidents (Lees et al., 2020, Conrod and Nikolaou, 2016).

Adolescence is a period of vital importance for the prevention of alcohol use and mental disorders (Sawyer et al., 2018, Kleinert and Horton, 2016). Throughout adolescence, there is an increased susceptibility for the development of alcohol use problems and mental ill-health (Costello et al., 2011). Even small increases in mental health symptoms in adolescence can increase the likelihood of developing a mental disorder as an adult (Fergusson, 2005). Further, evidence suggests that early onset of alcohol use increases the risk of subsequent heavy alcohol use and comorbid mental health problems (Teesson, 2005, Liang and Chikritzhs, 2015, Silins et al., 2018). Nationally representative interviews in the US have estimated that 50 – 70% of adults with a mental disorder experienced a disorder in their youth, based on retrospective accounts (Costello et al., 2011, Kessler et al., 2005). However, mental disorders are often undetected and untreated in young people due to problems recognising mental health problems and barriers to help-seeking (ten Have et al., 2013, Thornicroft, 2012, Wang et al., 2007).

Unfortunately, the effectiveness of existing prevention programs for alcohol use and mental disorders among adolescents tends to be modest and they suffer from implementation barriers (Fazel et al., 2014). School-based prevention programs tend to be universal, meaning that those with low risk of problematic alcohol use and mental disorders are given the same intervention as those at higher risk. Further, prevention programs typically target single disorders, meaning that multiple programs would be required to make an impact. *Preventure* is the only evidence-based prevention program addressing shared risk factors for problematic alcohol use, internalising problems and externalising problems in schools (Teesson et al., 2017, Conrod et al., 2006, Conrod et al., 2008, Newton et al., 2016, Newton et al., 2019, Castellanos and Conrod, 2006). *Preventure* is selectively delivered to adolescents with one of four personality risk factors: anxiety sensitivity, hopelessness, sensation seeking, and impulsivity. While *Preventure* was designed as a substance use prevention program, the four personality risk factors have been found to predict internalising and externalising problems in addition to problematic alcohol use and illicit drug use (Newton et al., 2016 2016/02/01/;53:23–30., Castellanos-Ryan et al., 2013, Woicik et al., 2009).

The effectiveness of the *Preventure* program when delivered by external psychologists has been demonstrated in Canada and the United Kingdom (Conrod et al., 2006, Conrod et al., 2008, Conrod et al., 2010, Castellanos and Conrod, 2006). A recent Australian cluster RCT replicated these results, indicating successful adaptation of the *Preventure* program for students in Australia when led by psychologists; results showed that *Preventure* successfully reduced the uptake of alcohol, binge drinking and alcohol-related harms up to 36-months following the intervention (Teesson et al., 2017, Newton et al., 2016, Newton et al., 2019, Barrett et al., 2015), with effect sizes (in Cohen’s *d*) of 0.47, 0.65 and 0.54 respectively (Conrod, 2016), equating to a moderate effect. In addition, the Australian adaptation showed secondary effects for mental health outcomes, including anxiety and depressive symptoms, conduct problems and hyperactivity symptoms (Newton et al., 2019).

### 1.1 Important next steps to advance prevention

Recent systematic reviews have highlighted a number of important areas for improving the evidence base on the prevention of alcohol use and mental disorders; a key recommendation is evaluating programs that target disorders with shared underlying vulnerabilities (Bennett et al., 2015, Stockings et al., 2016a, Stockings et al., 2016b). There is increasing evidence that *Preventure* is efficacious for internalising and externalising problems in addition to alcohol use (Newton et al., 2019, Castellanos and Conrod, 2006, Kelly et al., 2020, O'Leary-Barrett et al., 2013); strengthening this evidence base will address this gap in current prevention efforts.

Another important step in improving the field of prevention is to move from simply demonstrating efficacy to evaluating the translation and implementation of efficacious programs into wider use in the population (Gottfredson et al., 2015). Effectiveness trials seek to test whether the positive effects observed in efficacy trials are robust to variations to the intervention, population, setting, and other adaptations likely to occur when an intervention is implemented more broadly (Gottfredson et al., 2015). One UK trial shows evidence that a teacher-led delivery of *Preventure* demonstrated significant impacts on alcohol use and mental health outcomes, with effect sizes similar to those found when the intervention was delivered by external psychologists (O'Leary-Barrett et al., 2013, O'Leary-Barrett et al., 2010). The current study is an Australian-first trial to evaluate the effectiveness of the *Preventure* program when implemented by school staff in Australia. Confirming the effectiveness of the *Preventure* program when delivered by school staff will enhance the scalability of the program, by providing a more feasible delivery method for schools.

## 2 Objectives

The main aim of the current study is to examine the effectiveness of *Preventure* in preventing the onset or escalation of alcohol use, internalising problems and externalising problems by targeting shared personality risk factors, when delivered by school staff (e.g. teachers, school counsellors) in Australia. It is hypothesised that compared to ‘high-risk’ students (i.e. those scoring one standard deviation above the population mean for one of the four personality risk factors) in control schools, ‘high-risk’ students in *Preventure* schools will show significantly reduced:

i) Alcohol use (uptake, quantity and frequency, binge drinking), intentions to use alcohol, and alcohol-related harms;
ii) Internalising problems (anxiety and depressive symptoms); and
iii) Externalising problems (conduct problems and hyperactivity symptoms).

An additional aim of the study is to examine the implementation fidelity, feasibility and acceptability of the program when delivered by school staff.

## 3 Methods and analysis

**Fig. 1:**
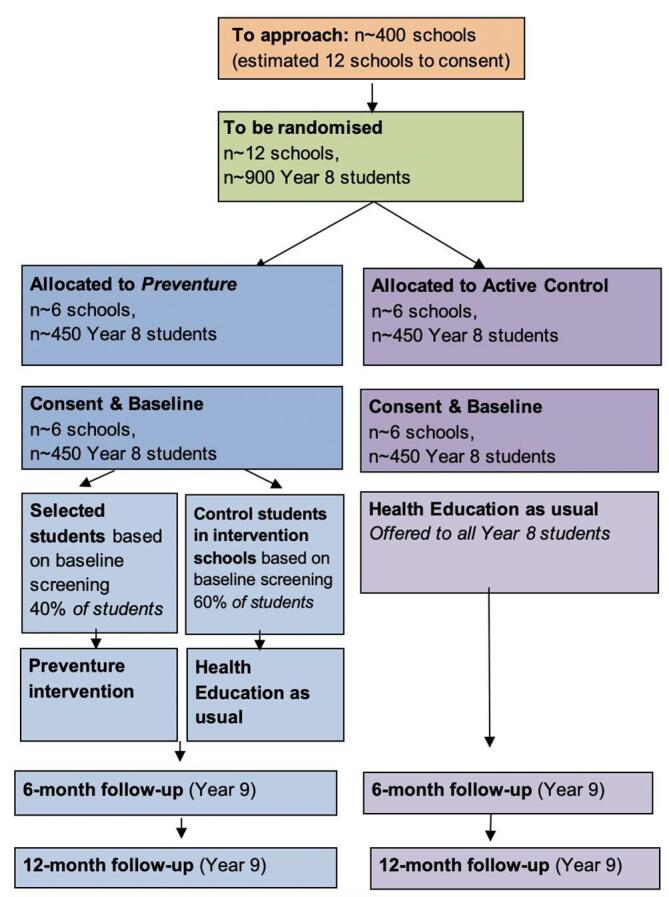
Flowchart of planned recruitment, randomisation and assessment process for 2020–2022, Sydney, Australia.

The School-led *Preventure* trial is a CONSORT-compliant cluster randomised controlled trial, to be conducted in 12 secondary schools in greater Sydney in NSW, Australia. Schools will be stratified by majority gender (i.e. >60% male or female) and randomised to either i) intervention (*Preventure*) or ii) control (health education as usual). Each condition will include 6 schools. *See* Fig. 1.

### 3.1 Inclusion criteria

Participants must be a Year 8 student (aged approximately 13 years) in a participating school at the time of randomisation and fluent in English. Teachers may exclude students at their discretion if they believe a student will not be able to undertake the program due to learning difficulties, or if they will be disruptive to other students. Any such students will be offered alternatives, such as one-on-one delivery. This will be recorded in staff fidelity measures.

Trial sites (schools) must:

i) Be a secondary or combined primary and secondary school in greater Sydney, NSW; and
ii) Have school principal permission to participate in the trial.
iii) Have at least 60 students enrolled in Year 8 at recruitment, to ensure there are sufficient group sizes to run the intervention.

### 3.2 Intervention

Schools allocated to the *Preventure* intervention will identify students to participate in the intervention based on their scores on the Substance Use Risk Profile Scale (SURPS; Woicik et al., 2009). Students scoring greater than one standard deviation above the population mean, as determined in the previous Australian trial (Newton et al., 2016), on any one of the traits measured by the SURPS (anxiety sensitivity, hopelessness, sensation seeking and impulsivity) will be invited to participate in the intervention that corresponds to the personality trait on which they score furthest from the mean. These groups are mutually exclusive: students can only participate in one personality-matched group. Students with elevated scores on more than one subscale will be allocated to the personality group where they deviated most from the population mean, according to z scores. Based on previous trials and Australian norms (Newton et al., 2016), it is anticipated that approximately 40% of students will screen into the intervention, with approximately equal numbers in each of the four groups.

The *Preventure* intervention consists of two 90-minute sessions with small groups of students (approximately 4–8 students per group), led by a trained facilitator and co-facilitator. Aided by real life example scenarios, the sessions incorporate: i) psycho-education about the target personality style and associated maladaptive coping behaviours (e.g., avoidance, aggression, and alcohol use); ii) development of an individualised model of the physical, cognitive and behavioural components of typical responses; and iii) motivational enhancement, goal-setting and application of cognitive-behavioural skills to modify problematic reactions and behaviours. At the end of each session, staff involved in the program delivery will be asked to complete a short survey measuring their adherence to the intervention as a measure of fidelity. The intervention will be delivered in addition to the NSW state-mandated Health and Physical Education curriculum for all Year 8 students. This curriculum includes lessons on mental health and wellbeing and alcohol and other drugs. Information on the health education as usual lessons will be made available to the research team by school staff.

### 3.3 School staff training and supervision

The facilitators and co-facilitators will include school staff, who will be trained according to the training protocol as described in O'Leary-Barrett et al. (2013). This includes a training workshop, followed by supervision to ensure adherence to the intervention. Supervision will include being observed by the trainer delivering the two 90-minute workshops for one personality type, with a group of students not included in the trial, and one hour of feedback from the trainer. Training and supervision can be delivered online if required due to Covid-19 restrictions. All facilitators will be provided with intervention manuals to assist in their delivery of the program (Table 1).

**Table 1:** Anticipated participant activities and assessment timeline for 2020–2022, Sydney, Australia.

Timing	September–December 2020	October 2020–March 2021	April–September 2021	October 2021–March 2022	January–July 2022
Participant					
Intervention staff	Training workshop + supervision	Preventure Intervention and intervention adherence survey	Staff survey		
Intervention students		1. Student baseline survey 2. Preventure intervention and feedback survey	Student survey 2: 6-month follow up	Student survey 3: 12-month follow up	
Control staff					Training workshop + supervision (offered)
Control students		Student baseline survey	Student survey 2: 6-month follow up	Student survey 3: 12-month follow up	

### 3.4 Control condition

Control schools will implement health and physical education as usual. The Year 8 Health and Physical Education curriculum mandates that mental health and wellbeing, and alcohol and other drug content be implemented. At the end of the study control schools will be informed of the results of the trial and offered complimentary use of the intervention, including training in *Preventure* delivery.

### 3.5 Outcomes

The primary outcomes are differences in the prevalence and/or means of ‘high-risk’ students in the intervention and control schools on alcohol use, internalising problems (anxiety and depressive symptoms) and externalising problems (conduct problems and hyperactivity symptoms), at 6- and 12-months post baseline. Specifically, it is hypothesised that ‘high-risk’ students in the intervention group will have lower prevalence/scores than ‘high-risk’ students in the control group, on the following primary outcome measures:

i) Alcohol use

A series of questionnaires adapted from the School Health and Alcohol Harm Reduction Project ‘Patterns of Alcohol’ index (McBride et al., 2004) will be used to measure intention to try alcohol, any use of alcohol ever (at least a standard drink), the frequency and quantity of alcohol consumption in standard drinks in the past six months, and frequency of binge drinking (five or more standard drinks on one occasion) in the past six months. Alcohol-related harms will be measured with an abridged form of the Rutgers Alcohol Problem Index (RAPI) (White and Labouvie, 1989). The original scale shows high internal consistency with adolescent samples (White and Labouvie, 1989). These questions were used in the previous Australian trial of *Preventure* (Newton et al., 2016, Newton et al., 2012).

ii) Internalising problems

The ‘Kessler 6′ scale (K6) (Kessler et al., 2002) will be used to assess psychological distress in the past month. The K6 shows high accuracy, consistency across samples, and specificity (Kessler et al., 2002). Depressive symptoms will be measured using the ‘Patient Health Questionnaire, modified for adolescents (PHQ-A) (Johnson et al., 2002), which demonstrates good sensitivity and specificity, diagnostic validity and accuracy (Johnson et al., 2002). Anxiety symptoms will be measured by the ‘Generalised Anxiety Disorder 7-item scale’ (GAD-7), which shows strong reliability and validity (Spitzer et al., 2006).

iii) Externalising problems

The conduct problems and hyperactivity subscales of the Strengths and Difficulties Questionnaire (SDQ) (Goodman, 1997) will be used to assess externalising problems. These subscales have been confirmed with a five-factor structure demonstrating satisfactory reliability and validity, predicting an increased likelihood of psychopathology (Goodman, 2001).

Secondary effects of Preventure will be explored for:

• Social anxiety [adapted from the Mini-Social Phobia Inventory (Mini-SPIN) (Connor et al., 2000)] and panic attacks (adapted from the Panic Attack Questionnaire (Norton et al., 2008);
• Other drug use (use of tobacco, e-cigarettes, and cannabis in the past six months);
• Bullying (adapted version of the Olweus Bully/Victim Questionnaire (Olweus, 1996);
• Emotion regulation (The Difficulties in Emotion Regulation Scale (Kaufman et al., 2016);
• School engagement (The Hemingway Measure of Adolescent Connectedness (Karcher and Lee, 2002));
• Quality of life (Satisfaction with Life Scale – Child version (Gadermann et al., 2010); and
• Self-compassion (Self-compassion scale: Short-form (Raes et al., 2011).

Implementation acceptability, feasibility, and fidelity outcomes

The following outcomes will be used to inform efforts to scale-up the delivery of *Preventure* across Australia.

• School staff-perceived feasibility and acceptability of delivering the *Preventure* intervention. Questions were developed for the trial using the RE-AIM framework (Glasgow et al., 1999) and seek information as to the perceived reach, effectiveness, adoption, implementation and maintenance of *Preventure* by school staff.
• Stakeholder views and an indication of any challenges or barriers associated with delivery of *Preventure* and selective prevention more broadly.
• Student feedback on the intervention, collected through a mix of open and closed questions about what they liked/disliked about the program
• Staff implementation fidelity (i.e. adherence to the intervention), collected via a self-report form completed by the main facilitator and a report made by the co-facilitator, assessing which parts of the intervention were covered, whether the facilitator made any changes to the intervention and the facilitator’s use of counselling techniques.

A range of other measures will also be included to describe the sample and explore the relationships between these measures and the primary outcomes:

• Demographics (age, gender, sex at birth, country of birth, grades at school, truancy, and a proxy for family socio-economic status used in the HBSC study, which includes items such as the number of cars and computers in the family home (Currie et al., 1997);
• Age of first alcohol consumption, maximum number of standard drinks consumed on one occasion and perceived proportion of students’ friends who drink alcohol (adapted from the School Health and Alcohol Harm Reduction Project ‘Patterns of Alcohol’ index (McBride et al., 2004);
• Other drug use (ecstasy, methamphetamine);
• Social contact and support (Items used from the National Survey on Mental Health (Slade et al., 2007);
• Interpersonal emotion regulation (a shortened version of the Interpersonal Regulation Questionnaire (Williams et al., 2018);
• Emotional neglect (adapted from the CDC-Kaiser Permanente Adverse Childhood Experiences Study (Felitti et al., 1998);
• Relationship with a parent/guardian (The Parental Bonding Instrument (Parker et al., 1979);
• Climate change anxiety (adapted from the Anticipatory Traumatic Reaction feelings subscale (Hopwood et al., 2017);
• Covid-19 exposure and impact (sourced from the Australian National Covid-19 mental health, behaviour and risk communication survey (Australian National University, 2020), and the Distress Questionnaire-5 (Batterham et al., 2016).

### 3.6 Sample size

This trial is powered to detect differences between ‘high-risk’ students (i.e. the students scoring one standard deviation above the population mean on one of the four personality types on the SURPS) in control schools vs ‘high-risk’ students in intervention schools. A total of 280 ‘high-risk’ students from 10 schools are required (i.e. 28 ‘high-risk’ students per school and 5 schools per intervention group) to achieve 80% power to detect a standardized between-group mean difference of 0.3 (p = .05) in outcomes. A between-group effect size of 0.3 is in line with effect sizes observed in previous *Preventure* trials (Newton et al., 2016). To account for dropouts, we aim to recruit 12 schools at baseline (6 per group) with 75 consenting students per school, 40% of whom are expected to screen into the program, based on previous research (Conrod et al., 2008), giving a total of 360 ‘high-risk’ students to test the effect of the intervention.

### 3.7 Recruitment and consent

All eligible schools in greater Sydney will be approached using publicly available contact details. The study will also be promoted to schools through professional networks. School Principals will be sent an invitation letter via email describing the study and seeking permission to implement the study in their school. Schools will be followed up with phone calls to discuss enrolment. Principals who agree for their school to participate, and who are randomised to the intervention group, will identify two to four staff members who may be interested in participating in the trial. Staff members will receive further information about the trial, and if they decide to participate, they will be asked to complete consent forms, agreeing to undertake the *Preventure* training, coordinate information and consent procedures, deliver the *Preventure* groups, and complete an evaluation survey of the *Preventure* program and its implementation.

Once the schools have been randomised, they will be sent the information and consent forms for parents/guardians of Year 8 students in the school, and will be asked to send these to the parents/guardians via their usual communication method. Students who receive active parental consent (opt in) must also provide their own consent before beginning the study. Participants are informed that they are free to withdraw from the study at any time.

### 3.8 Allocation

Blocked randomisation of schools to *Preventure* and control, stratified by school gender mix [co-educational, predominately male (>60%), or predominately female (>60%)], will be performed by an external researcher using the Blockrand package in R (Snow, 2020). Allocation concealment is ensured as the external researcher does not release the randomisation until the school has been irreversibly allocated to a condition. Schools will not be masked to their allocation and will be informed whether they are in the intervention or control group. Participants (i.e. students) will be blinded to the hypotheses of the study. As is the case for school-based interventions of this kind, students, teachers, and researchers will be aware of the schools’ allocation.

### 3.9 Data collection methods and data management

All students (intervention and control conditions) will be asked to complete an online self-report survey in class at baseline (prior to the intervention), and 6- and 12-months post-baseline. Students will enter a unique identifier and will enter this code each time they complete a questionnaire. The student’s data files will be linked over time with this unique code, whilst maintaining confidentiality. Responses to the SURPS (Woicik et al., 2009) will be linked to this unique code; scores on the SURPS will be used to classify students into groups, and once classified the codes will be re-identified in a separate database and sent to the school contact in a secure link. Contact details will be marked as identifiers and access restricted. Students will be made aware that all contact information will be kept confidential and secure, separate from their survey responses.

School staff in the intervention condition will complete an online survey to collect the implementation outcomes (e.g., feasibility, acceptability) of *Preventure.* The survey contains a mix of closed and open-ended questions. Intervention students and staff will also complete a brief paper-based evaluation survey at the end of the last workshop. A paper-based survey was chosen for ease of completion immediately post-group. For students, the evaluation survey includes both open and closed questions, such as what they liked/disliked about *Preventure* and whether the information covered was helpful. For staff, the evaluation survey will measure implementation fidelity, that is, their adherence to the intervention. No identifying information will be collected in these surveys. All data collected on paper surveys will be entered into an electronic database on the University of Sydney server.

To maximise retention, students who are absent on the days of data collection will be contacted by school staff to offer an alternate time to complete the survey. Additionally, student participants will be entered into a prize draw to win a $50 gift voucher for each survey they complete. Staff in both intervention and control schools will receive a reimbursement of $50 for their time at the conclusion of the study.

### 3.10 Statistical methods

Intention-to-treat analyses will be carried out for all primary and secondary outcomes, among the ‘high-risk’ students. Baseline equivalence and attrition between groups will be examined using single-level analyses; one-way analyses of variance (normally distributed data), chi-squared analyses (binominal data), and Mann-Whitney U-tests (non-normally distributed data). To examine intervention by time interaction effects, mixed effects regression will be used due to the multi-level nature of the data (students nested within schools). Hypothesised intervention effects on alcohol use, internalising problems and externalising problems will be examined using mixed effects linear regression analysis (continuous data) and mixed effects logistic regression analysis (categorical data). Missing data will be handled by full information maximum likelihood, in accordance with the intention-to-treat principle, which includes all randomised participants. Data from the school staff survey will be predominantly descriptive.

### 3.11 Monitoring

During school staff training and *Preventure* delivery, schools’ duty of care procedures will be reviewed by the investigators, to ensure adequate care should students feel distress while completing the survey or during the intervention. In addition, should students disclose current risk of harm during the survey, a notification will be sent to members of the research team. Should this occur, the project coordinator will re-identify the participant’s name and notify the relevant school counsellor/other nominated staff member to request that they follow up with the student. From this point the school’s usual duty of care procedure will be followed.

### 3.12 Ethics and dissemination

Ethical approval has been provided by the University of Sydney Human Research Ethics Committee, approval number 2019/792, and by the State Education Research Applications Process, the approval body for research in Public (State) schools in NSW. The Cochrane Risk of Bias tool will be used in disseminating the results of this trial (Sterne et al., 2019).

## 4 Discussion

Alcohol use and mental disorders cause substantial harm to young people and impact the wider society (Vigo et al., 2016). To reduce the cost and burden of alcohol use and mental disorders, timely and effective prevention is critical. This paper describes the protocol for the School-led *Preventure* study, an Australian-first effectiveness trial to test delivery of a prevention program targeting shared risk factors for problematic alcohol use, internalising problems and externalising problems, with the potential to be rolled out across Australia at relatively low cost.

### 4.1 Strengths and limitations

A major strength of this study is the progression from examining efficacy, to an examination of effectiveness and feasibility when implemented by school staff in the classroom. It is essential that schools are able to deliver evidence-based programs for preventing problematic alcohol use and mental ill-health that are practicable. *Preventure* is a brief and low-cost program, that can be incorporated into the school curriculum, addressing core components of PDHPE (Edalati and Conrod, 2019, Miller, 2008). Moreover, upskilling existing school staff already embedded within the school, rather than relying on external psychologists, has the potential to further reduce costs and increase longevity of program implementation. By targeting personality factors that are drivers of alcohol use, internalising problems and externalising problems, the *Preventure* program can efficiently address multiple emotional and behavioural problems in two 90-minute sessions. Further, this study has the potential to broaden the evidence base of the *Preventure* program, beyond alcohol use and mental health, to other important areas of adolescent wellbeing.

The current study is not without limitations. As with most research examining adolescent substance use, our outcome measures are reliant on student self-report. As students will be disclosing illegal behaviour, their responses may be subject to bias. However, studies have demonstrated self-report to be a valid method of assessing adolescent substance use symptoms (Needle et al., 1983). Additionally, students are informed their responses are confidential, thereby reducing the risk of concealment or bias.

In addition, given the longitudinal study design there is the potential for participant attrition. Further, the training and program delivery require a significant amount of staff time, which may impact on recruitment and retention, particularly given the impact of the Covid-19 pandemic on schools. However, at the time of writing, schools in Sydney are conducting classroom learning, and to maximise recruitment and retention, schools will be offered a flexible training and implementation schedule. Furthermore, by contacting students who may be absent on days of follow-up data collection, we hope to minimise participant attrition.

### 4.2 Implications

Delivery of evidence-based programs for problematic alcohol use and mental ill-health in the school setting is limited. There are several barriers to implementation, such as cost, geographic restrictions, and access to training. Importantly, this trial aims to improve access to an innovative, evidence-based program, by upskilling school staff to lead the intervention themselves. Further, the findings of this trial could be used to strengthen the evidence base for the *Preventure* program in addressing multiple problems in one brief intervention, and support a scalable and sustainable model of delivery.

## Sponsor and funder

The funder for this trial has no input into the study design or data collection, management, analysis or interpretation. The funding has no role in the decision to submit results for publication.

## Funding

This work was supported by an Australian Rotary Health Mental Health of Young Australians Research Grant to Dr Erin Kelly.

## Author contributions

EK, NN, LB, LS, EB, and MT conceptualised, designed and obtained funding for the study. LG, EK, LB, LS, EB and NN obtained ethical approval for the study, with the assistance of all authors. JB, LG and EK are responsible for recruitment, with assistance from MT and LS. EK, LG, LB, NN, EB, LS and MT drafted the manuscript, and all authors approved the manuscript.

## Declaration of Competing Interest

The authors declare that they have no known competing financial interests or personal relationships that could have appeared to influence the work reported in this paper.
