# Supplementary material for: The school-led Preventure study: Protocol of a cluster-randomised controlled trial of effectiveness to prevent adolescent alcohol misuse, internalising problems, and externalising problems through a personality-targeted intervention delivered by school staff
Source: Prev Med Rep. 2020 Dec 19;21:101286. doi: 10.1016/j.pmedr.2020.101286 (PMC7772564; doi:10.1016/j.pmedr.2020.101286)
Supplement: Supplementary data 2 [file mmc2.docx]

**
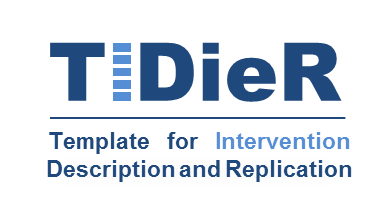
The TIDieR (Template for Intervention Description and Replication) Checklist*:**

Information to include when describing an intervention and the location of the information

| **Item number** | **Item** | **Where located **** |
| --- | --- | --- |
|  |  | Primary paper  (page or appendix  number) |
|  | **BRIEF NAME** | Conrod 2016 [1] |
| **1.** | Provide the name or a phrase that describes the intervention.  Preventure |  |
|  | **WHY** | Conrod 2016 [1] |
| **2.** | Describe any rationale, theory, or goal of the elements essential to the intervention.  Targeting personality traits linked to adolescent substance misuse and psychopathology, using cognitive-behavioural and motivational interviewing techniques. |  |
|  | **WHAT** |  |
| **3.** | Materials: Describe any physical or informational materials used in the intervention, including those provided to participants or used in intervention delivery or in training of intervention providers. Provide information on where the materials can be accessed (e.g. online appendix, URL).  Facilitator manuals providing to staff leading the intervention, student manuals provided to all students. Three core components were involved: psychoeducation; behavioural coping skills; cognitive coping skills. | These materials are copyrighted and cannot be accessed publicly. |
| **4.** | Procedures: Describe each of the procedures, activities, and/or processes used in the intervention, including any enabling or support activities. | See trial registry  [ANZCTR 380016](https://www.anzctr.org.au/Trial/Registration/TrialReview.aspx?id=380016&isReview=true) |
|  | **WHO PROVIDED** |  |
| **5.** | For each category of intervention provider (e.g. psychologist, nursing assistant), describe their expertise, background and any specific training given. | Preventure training protocol as per [2] |
|  | **HOW** |  |
| **6.** | Describe the modes of delivery (e.g. face-to-face or by some other mechanism, such as internet or telephone) of the intervention and whether it was provided individually or in a group. | 2 x face-to-face group sessions |
|  | **WHERE** |  |
| **7.** | Describe the type(s) of location(s) where the intervention occurred, including any necessary infrastructure or relevant features. | School classroom |
|  | **WHEN and HOW MUCH** |  |
| **8.** | Describe the number of times the intervention was delivered and over what period of time including the number of sessions, their schedule, and their duration, intensity or dose. | Participants received 2 x 90 minute sessions over a fortnight. |
|  | **TAILORING** |  |
| **9.** | If the intervention was planned to be personalised, titrated or adapted, then describe what, why, when, and how. | 4 separate interventions are delivered, specific to each personality type targeted (anxiety sensitivity, negative thinking, sensation seeking, impulsivity). |
|  | **MODIFICATIONS** |  |
| **10.^ǂ^** | If the intervention was modified during the course of the study, describe the changes (what, why, when, and how). | N/A |
|  | **HOW WELL** |  |
| **11.** | Planned: If intervention adherence or fidelity was assessed, describe how and by whom, and if any strategies were used to maintain or improve fidelity, describe them. | Facilitator self-report, co-facilitator report of facilitator |
| **12.^ǂ^** | Actual: If intervention adherence or fidelity was assessed, describe the extent to which the intervention was delivered as planned. | N/A |

** **Authors** - use N/A if an item is not applicable for the intervention being described. **Reviewers** – use ‘?’ if information about the element is not reported/not sufficiently reported.

† If the information is not provided in the primary paper, give details of where this information is available. This may include locations such as a published protocol or other published papers (provide citation details) or a website (provide the URL).

ǂ If completing the TIDieR checklist for a protocol, these items are not relevant to the protocol and cannot be described until the study is complete.

* We strongly recommend using this checklist in conjunction with the TIDieR guide (see *BMJ* 2014;348:g1687) which contains an explanation and elaboration for each item.

* The focus of TIDieR is on reporting details of the intervention elements (and where relevant, comparison elements) of a study. Other elements and methodological features of studies are covered by other reporting statements and checklists and have not been duplicated as part of the TIDieR checklist. When a **randomised trial** is being reported, the TIDieR checklist should be used in conjunction with the CONSORT statement (see [www.consort-statement.org](http://www.consort-statement.org)) as an extension of **Item 5 of the CONSORT 2010 Statement.** When a **clinical trial** **protocol** is being reported, the TIDieR checklist should be used in conjunction with the SPIRIT statement as an extension of **Item 11 of the SPIRIT 2013 Statement** (see [www.spirit-statement.org](http://www.spirit-statement.org)). For alternate study designs, TIDieR can be used in conjunction with the appropriate checklist for that study design (see [www.equator-network.org](http://www.equator-network.org)).

1. Conrod PJ. Personality-Targeted Interventions for Substance Use and Misuse. Current addiction reports. 2016;3(4):426-36. PubMed PMID: 27909645. Epub 2016/11/04. eng.

2. O'Leary-Barrett M, Topper L, Al-Khudhairy N, Pihl RO, Castellanos-Ryan N, Mackie CJ, et al. Two-Year Impact of Personality-Targeted, Teacher-Delivered Interventions on Youth Internalizing and Externalizing Problems: A Cluster-Randomized Trial. Journal of the American Academy of Child & Adolescent Psychiatry. 2013 Sep;52(9):911-20. PubMed PMID: 2013-30818-010. English.
